# Cost and efficacy comparison of prenatal recall and reflex DNA screening for trisomy 21, 18 and 13

**DOI:** 10.1371/journal.pone.0220053

**Published:** 2019-07-25

**Authors:** Jonathan Paul Bestwick, Nicholas John Wald

**Affiliations:** Wolfson Institute of Preventive Medicine, Barts and the London School of Medicine and Dentistry, Queen Mary University of London Charterhouse Square, London, United Kingdom; University of Cambridge, UNITED KINGDOM

## Abstract

**Objective:**

To compare costs and efficacy of reflex and recall prenatal DNA screening for trisomy 21, 18 and 13 (affected pregnancies). In both methods women have Combined test markers measured. With recall screening, women with a high Combined test risk are recalled for counselling and offered a DNA blood test or invasive diagnostic testing. With reflex screening, a DNA analysis is automatically performed on plasma collected when blood was collected for measurement of the Combined test markers.

**Methods:**

Published data were used to estimate, for each method, using various unit costs and risk cut-offs, the cost per woman screened, cost per affected pregnancy diagnosed, and for a given number of women screened, numbers of affected pregnancies diagnosed, unaffected pregnancies with positive results, and women with unaffected pregnancies having invasive diagnostic testing.

**Results:**

Cost per woman screened is lower with reflex v recall screening: £37 v £38, and £11,043 v £11,178 per affected pregnancy diagnosed (DNA £250, Combined test markers risk cut-off 1 in 150). Reflex screening results in similar numbers of affected pregnancies diagnosed, with 100-fold fewer false-positives and 20-fold fewer women with unaffected pregnancies having invasive diagnostic testing.

**Conclusions:**

Reflex DNA screening is less expensive, more cost-effective, and safer than recall screening.

## Introduction

Prenatal screening for trisomy 21 (Down syndrome), trisomy 18 (Edwards syndrome), and trisomy 13 (Patau syndrome) using plasma (cell-free) DNA analysis detects nearly all affected pregnancies (pregnancies with trisomy 21, 18 or 13) with a much lower positive rate (proportion of unaffected pregnancies with a positive screening result) compared with conventional screening methods based on the measurement of ultrasound and serum markers.[1,2] DNA testing has however not generally been implemented as a method of routine screening, because it is more complex and costly than conventional screening methods, and has a failure rate due to technical reasons or biological reasons, for example when the fetal fraction (percentage of cell-free DNA from the placenta) is low.[3] Two screening methods to overcome this have been proposed.

**Fig 1:**
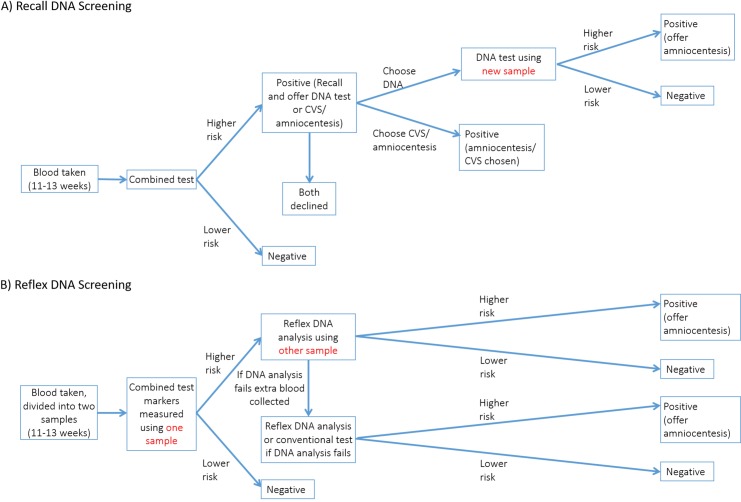
Flow diagram of Recall (A) and Reflex (B) DNA screening.

One method, “recall DNA screening” recommended by the UK National Screening Committee [4], involves women having a first trimester Combined test (based on the measurement of nuchal translucency, pregnancy associated plasma protein A, free beta human chorionic gonadotrophin, and maternal age). The 2–3% of women with positive Combined test results (risk of an affected pregnancy ≥1 in 150) are recalled for counselling and offered an invasive diagnostic test (amniocentesis or chorionic villus sampling) or having another blood sample collected for a DNA test. Women with a positive DNA test are then offered a diagnostic test. The recall method has a detection rate (proportion of affected pregnancies with a positive screening result) similar to that of conventional screening. [5,6] In one study [5] about 18% of women with a positive Combined test were sufficiently worried by their Combined test risk to choose an invasive diagnostic test without having a DNA test first. The recall DNA screening method is illustrated in Fig 1A.

The other method, “reflex DNA screening” involves collecting extra blood at the time blood is collected for measurement of the Combined test serum markers, and retaining a plasma sample for potential DNA analysis. If the woman has a risk of having an affected pregnancy based on the Combined test markers at or above a pre-specified cut-off a DNA test is automatically triggered using the retained sample (i.e. a reflex response to the Combined test markers risk estimate). This avoids having to recall women for counselling and avoids having to obtain an extra blood sample [7,8] thereby achieving a much reduced false-positive rate and an earlier DNA screening result compared with the recall method. In reflex DNA screening all women undergo a single test procedure and each woman receives a single screening result; with recall DNA screening this is not the case. All women have a Combined test and receive a screening result, and some women are invited for a second screening test (DNA test) and receive a second screening result. Reflex DNA screening has been implemented in routine practice. [9] The reflex DNA screening method is illustrated in Fig 1B.

We here compare the efficacy of reflex and recall DNA screening with their associated costs in a UK setting and provide an Online screening cost calculator that can be used to compare the two methods using other currency unit costs (e.g. cost of a DNA analysis) given that the unit costs will vary over time and from place to place.

## Materials and methods

We used published estimates of the costs [5] (Table 1) and estimates of screening performance [9] and uptake rates [5] according to Combined test markers risk cut-off (Table 2; see S1 Fig for the derivation of the estimates in Table 2 and the source of the data used) associated with each element of the recall and reflex methods to calculate the number of affected pregnancies (with trisomy 21, 18 or 13) diagnosed, number of false-positive results, number of invasive diagnostic tests performed in unaffected pregnancies, the overall cost of screening 100,000 women, the cost per woman screened and the cost per affected pregnancy diagnosed, for each of the two methods. We used the prevalence of affected pregnancies collectively (1 in 215) observed in practice [9] so that in 100,000 women there are 465 expected affected pregnancies. The screening performance of the DNA test was taken from an implementation project of reflex DNA screening [9]. Among pregnancies that were reflexed the detection rate of the DNA analysis was 98.1% (101/103) and the false-positive rate was 0.08% (2/2377). The uptake rate of chorionic villus sampling (CVS) or amniocentesis following a positive DNA test was taken as 90% [5]. We provide cost estimates based on specified term Combined test markers risk cut-offs from 1 in 150 to 1 in 800. We provide separate cost estimates using EDTA collection tubes and DNA stability tubes. Given that the cost of DNA testing is likely to come down in the future, cost estimates were also calculated using DNA test costs of £250, £200, £150 and £100.

**Table 1:** Input unit cost estimates*.

Item	Cost
Combined test	£27.52
Counselling screen positive women	£15.96
Recall blood sample collection and transportation	£9.00
DNA test	£250
Amniocentesis/CVS	£650

*Costs reported by Chitty et al [4]

**Table 2:** Input test performance and uptake estimates according to Combined test markers risk cut-off. For derivation of values see S1 Fig.

			Recall DNA screening only: Women's choices following a positive Combined test					
Combined test markers risk cut-off			Affected			Unaffected		
	Proportion ≥cut-off		Amniocentesis/CVS	DNA test	Decline further testing	Amniocentesis/CVS	DNA test	Decline further testing
	Affected	Unaffected						
1 in 150	81%	2.4%	46.2%	48.8%	5.0%	13.3%	78.4%	8.3%
1 in 200	84%	3.0%	44.5%	50.1%	5.4%	10.6%	80.9%	8.5%
1 in 300	90%	4.4%	41.5%	53.0%	5.5%	7.3%	83.9%	8.8%
1 in 400	91%	5.5%	41.1%	53.2%	5.7%	5.8%	85.2%	9.0%
1 in 600	93%	8.0%	40.3%	53.9%	5.8%	4.0%	86.9%	9.1%
1 in 800	97%	10.5%	38.6%	55.7%	5.8%	3.0%	87.7%	9.2%

## Results

Table 3 shows a comparison of the screening efficacy and costs between recall and reflex DNA screening according to Combined test markers risk cut-off levels and the cost of a DNA analysis. (S1 Table shows screening performance and costs for a greater range of Combined test risk markers cut-offs and DNA analysis costs). Reflex and recall DNA screening leads to the diagnosis of a similar number of affected pregnancies but with reflex DNA screening far fewer women with unaffected pregnancies are given a positive result and far fewer invasive diagnostic tests are performed in unaffected pregnancies. For example, in 100,000 women screened, using a Combined test markers risk cut-off of 1 in 150 more than 100-fold fewer women with unaffected pregnancies are given a positive test result (2389 v 19) and about 20-fold fewer women have an invasive diagnostic test (17 v 332). The cost per woman screened and the cost per affected pregnancy diagnosed are lower with reflex DNA screening than with recall DNA screening regardless of the Combined test markers risk cut-off and DNA analysis cost shown in Table 3 and S1 Table. For example, with a DNA analysis cost of £250 reflex DNA screening costs about £1 less per woman screened than recall DNA screening for all the specified Combined test markers risk cut-offs.

**Table 3:** Comparison of efficacy and costs of recall and reflex DNA screening according to Combined test markers risk cut-off and cost of the DNA analysis.

DNA test cost		Recall DNA screening with Combined test markers risk cut-off of:-				Reflex DNA screening with Combined test markers risk cut-off of:-			
		1 in 150	1 in 200	1 in 300	1 in 800	1 in 150	1 in 200	1 in 300	1 in 800
	In 100,000 women screened:								
	Affected pregnancies (trisomy 21, 18 and 13) prenatally diagnosed*	337	347	369	395	333	346	369	398
	False-positives	2389	2986	4380	10451	19	24	35	84
£250	Diagnostic tests in unaffected pregnancies	332	335	344	384	17	22	32	76
	Total cost	£3,767,146	£3,928,813	£4,305,275	£5,877,476	£3,677,208	£3,841,962	£4,219,252	£5,793,995
	Cost per woman screened	£38	£39	£43	£59	£37	£38	£42	£58
	Cost per affected pregnancy diagnosed	£11,178	£11,322	£11,667	£14,880	£11,043	£11,104	£11,434	£14,558
	In 100,000 women screened:														
	Affected pregnancies (trisomy 21, 18 and 13) prenatally diagnosed*	337	347	369	395	333	346	369	398
	False-positives	2389	2986	4380	10451	19	24	35	84
£100	Diagnostic tests in unaffected pregnancies	332	335	344	384	17	22	32	76
	Total cost	£3,458,446	£3,537,163	£3,720,725	£4,464,326	£3,262,308	£3,335,412	£3,499,552	£4,158,695
	Cost per woman screened	£35	£35	£37	£45	£33	£33	£35	£42
	Cost per affected pregnancy diagnosed	£10,262	£10,194	£10,083	£11,302	£9,797	£9,640	£9,484	£10,449

*The number of affected pregnancies diagnosed by the two methods differs as a result of two competing effects: (i) a proportion of women who have recall screening decline a DNA test or a diagnostic test following a positive Combined test result and (ii) a proportion of women who have recall DNA screening choose a diagnostic test following a positive Combined test result.

## Discussion

**Fig 2:**
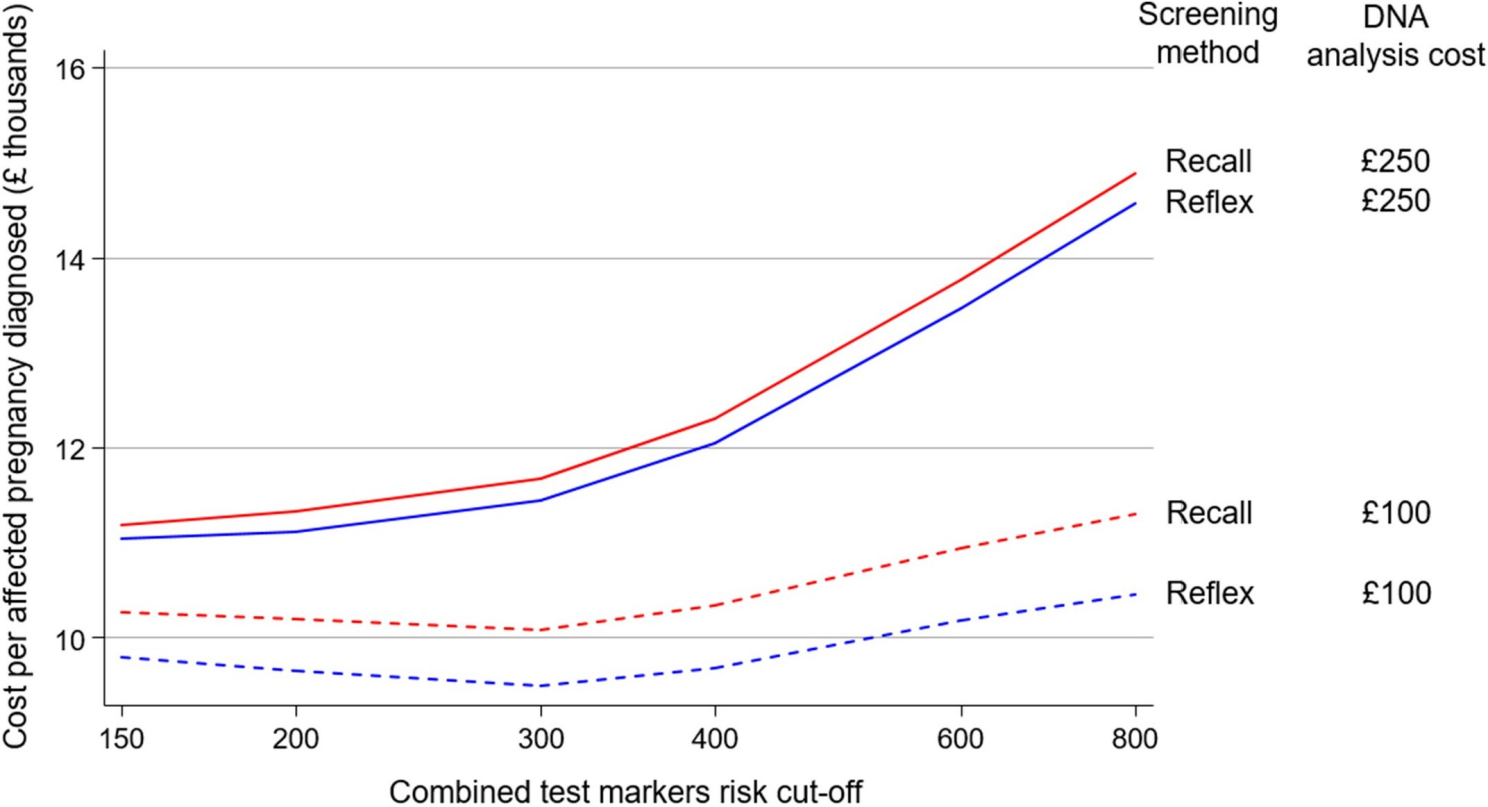
Cost per affected pregnancy (trisomy 21, 18 or 13) diagnosed according to the combined test markers risk cut-off used to select women for a DNA analysis for recall and reflex DNA screening and DNA analysis costs of £250 and £100.

Our results show that using published UK unit cost estimates, reflex DNA screening is less expensive and more cost-effective than the recall method and has over 100-fold lower false-positive results (see Fig 2). As the cost of the DNA analysis continues to decline, as is likely, a greater proportion of women can be reflexed to a DNA analysis with the associated increase in the detection rate while maintaining a low number of false-positive results and cost effectiveness (see Table 3), an advantage not achievable with the recall method. Costs vary from place to place and over time. For this reason, we have produced an online screening cost and efficacy calculator (www.screening-cost-calculator.com) that allows local unit costs to be entered (in £, $ or €) and local versions of Table 3 are generated (S2 Fig) and local programme costs determined for the two screening methods.

Our cost estimate of screening using the recall method with a 1 in 150 Combined test risk cut-off (£3.8 million per 100,000 women screened) is similar to the estimate in the study we used to obtain the component unit costs [5], taking into account that that estimate was based on women offered screening with 66% accepting (£3.7 million per 100,000 women screened)

The main contribution to the total cost for both the recall and reflex methods is from measuring the Combined test markers and calculating the risk of an affected pregnancy based on them, which all women receive. Although the DNA analysis is substantially more expensive, only a small proportion have a DNA analysis so it contributes less to the total cost. There may be additional costs for the recall method should women with a high Combined test risk but a negative DNA test result be sufficiently anxious about the Combined test risk to request and amniocentesis. However, this would only apply to a small number of women, and Chitty et al did not report any such women. [5].

Costs are just one element in determining policy. It cannot be ignored that the number of false-positives with the recall method is over 100 times greater than with the reflex method; a considerable burden of anxiety that the reflex method avoids (Table 3). The anxiety caused is well illustrated in an implementation study of the recall method that observed that 18% of the 2–3% of women recalled due to a high Combined test risk estimate requested an invasive diagnostic test without having a DNA analysis first [5], even though many of these pregnancies would be unaffected, the proportion being higher in affected pregnancies than unaffected pregnancies because they have, on average, higher Combined test risk estimates and are therefore more anxious (see Table 2). These unnecessary diagnostic procedures would be avoided with the reflex method, making it the safer screening method. The reflex method also avoids the time, inconvenience and costs to the women returning to the hospital or clinic for counselling that arise with the recall method.

Our estimates of costs were based on the use of EDTA blood collection to provide plasma for a DNA analysis from all women as is current practice in the Wolfson Institute antenatal screening service. The use of EDTA tubes is acceptable as there is evidence that the separation of plasma from cells up to a least 48 hours after blood collection does not significantly degrade the sample needed for DNA analysis [10,11] and unpublished data from the Wolfson Institute screening service indicate that this is the case for up to 60 hours. The use of DNA stability blood collection tubes would add significantly to the cost of reflex DNA screening. We have included a cost input item for this as an option to the screening cost and efficacy calculator. S2 Table, provides in a similar way to Table 3, cost estimates using DNA stability tubes. The added cost of DNA stability blood collection tubes when EDTA tubes can be used emphasises the importance of ensuring that blood samples are delivered to the DNA laboratory within 60 hours of collection.

In summary a reflex DNA screening programme is no more expensive than the recall method, is more effective, safe and more cost-effective than a programme based on the recall method.

## Supporting information

S1 Fig Derivation and source of values in Table 2 for a 1 in 150 Combined Test Markers (CTM) risk cut-off. For lower CTM risk cut-offs in Table 2 it is assumed the absolute number of women choosing a CVS/amniocentesis remains constant.
(TIF)

S2 Fig Online screening cost and efficacy calculator. **Available at** www.screening-cost-calculator.com
(TIF)

S1 Table Comparison of efficacy and costs of recall and reflex DNA screening according Combined test markers risk cut-off and cost of the DNA analysis. (DOCX)

S2 Table Comparison of efficacy and costs of recall and reflex DNA screening according to Combined test markers risk cut-off and cost of the DNA analysis: Cost calculations include the cost of DNA stability blood collection tubes. (DOCX)
