# Supplementary figures and images for: Cost and efficacy comparison of prenatal recall and reflex DNA screening for trisomy 21, 18 and 13
Source: PLoS One. 2019 Jul 25;14(7):e0220053. doi: 10.1371/journal.pone.0220053 (PMC6658079; doi:10.1371/journal.pone.0220053)

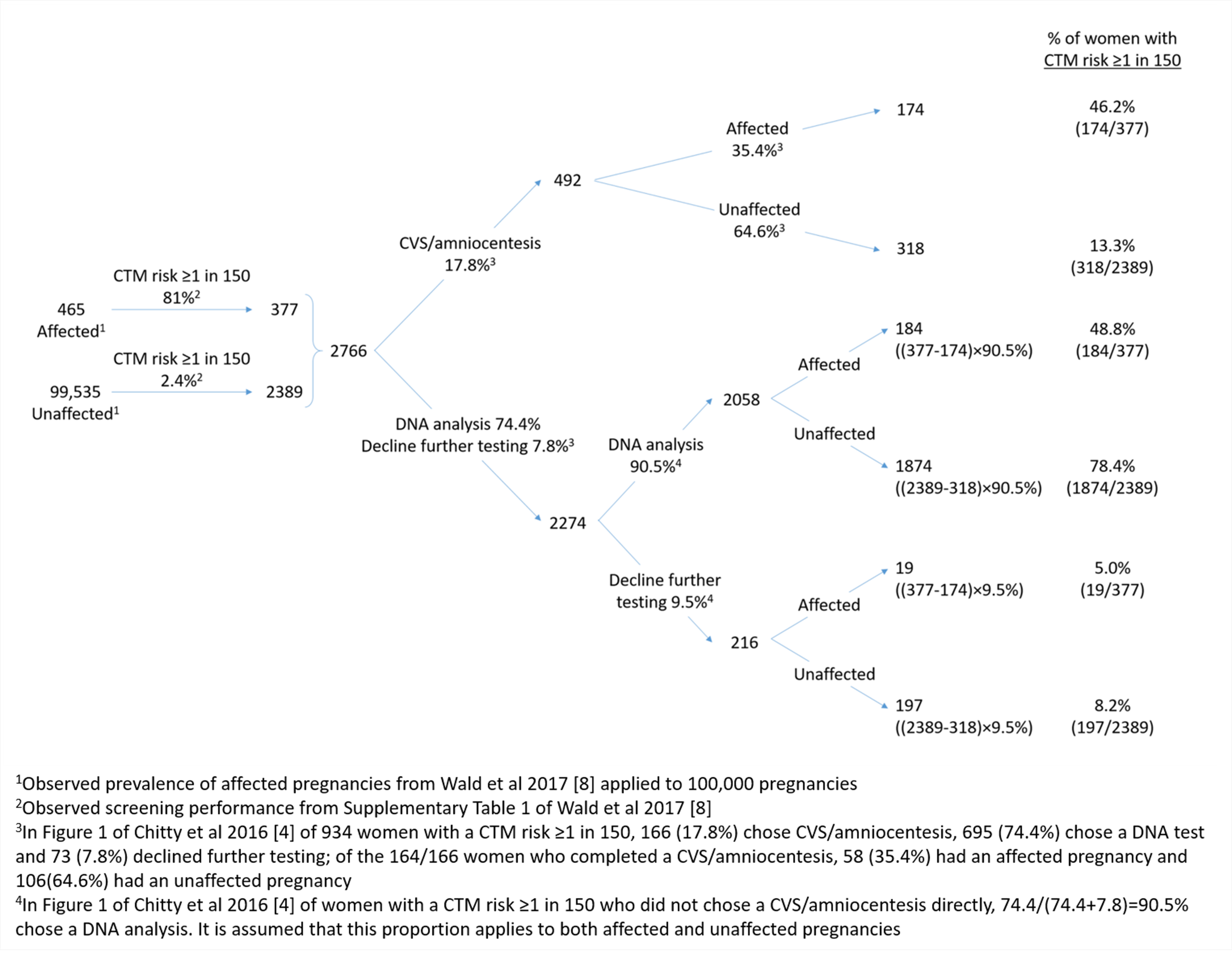

Supplement: S1 Fig — For lower CTM risk cut-offs in Table 2 it is assumed the absolute number of women choosing a CVS/amniocentesis remains constant. (TIF) [file pone.0220053.s001.tif]

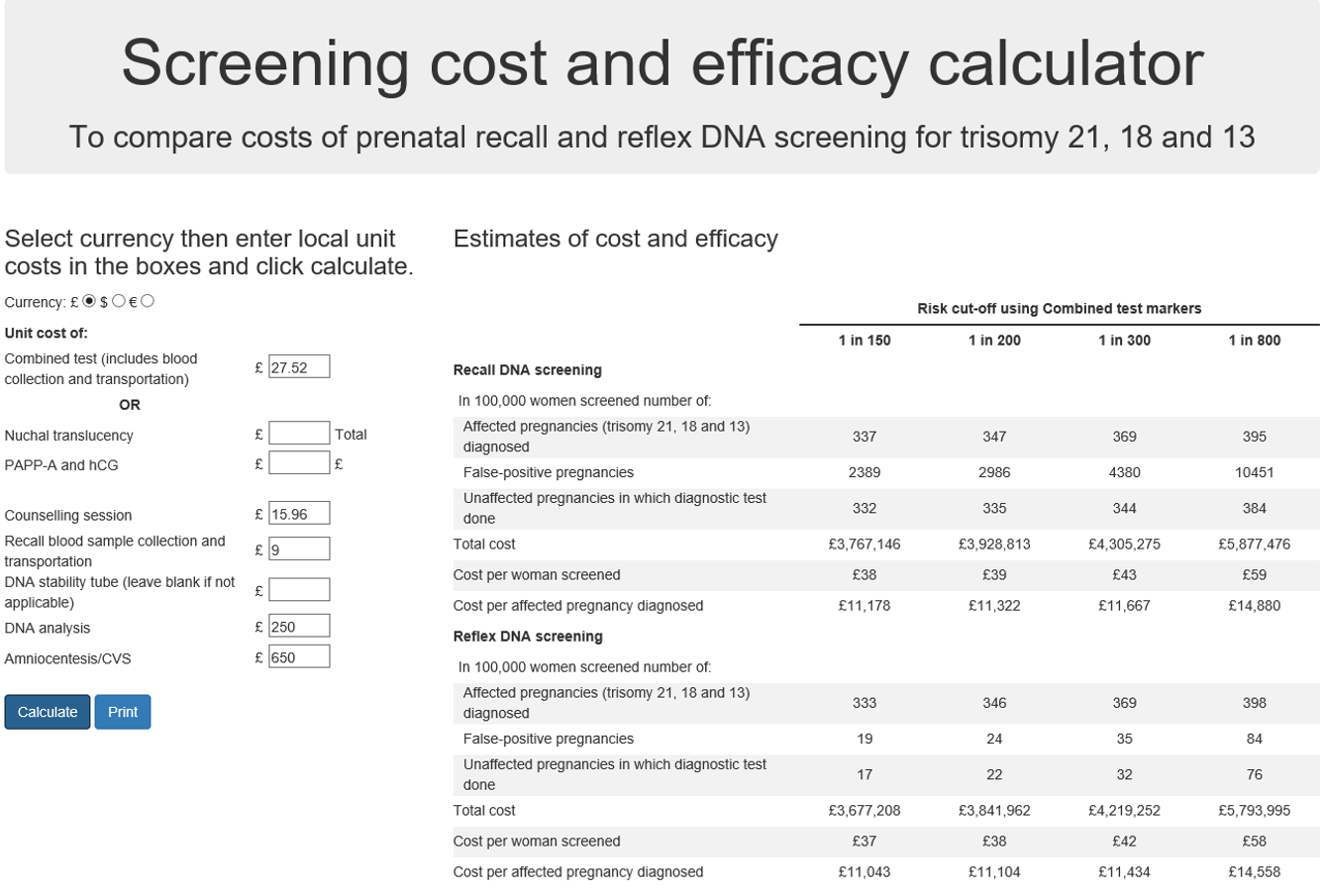

Supplement: S2 Fig — Available at www.screening-cost-calculator.com (TIF) [file pone.0220053.s002.tif]
