# Supplementary material for: Cost and efficacy comparison of prenatal recall and reflex DNA screening for trisomy 21, 18 and 13
Source: PLoS One. 2019 Jul 25;14(7):e0220053. doi: 10.1371/journal.pone.0220053 (PMC6658079; doi:10.1371/journal.pone.0220053)
Supplement: S1 Table — (DOCX) [file pone.0220053.s003.docx]

S1 Table. Comparison of efficacy and costs of recall and reflex DNA screening according Combined test markers risk cut-off and cost of the DNA analysis

*The number of affected pregnancies diagnosed by the two methods differs as a result of two competing effects: (i) a proportion of women who have recall screening decline a DNA test or a diagnostic test following a positive Combined test result and (ii) a proportion of women who have recall DNA screening choose a diagnostic test following a positive Combined test result
